# Evaluating the Link Between High‐Density Lipoprotein‐Related Inflammatory Indices and Gallstone Disease in U.S. Adults

**DOI:** 10.1155/mi/1925004

**Published:** 2026-02-04

**Authors:** Chang Fu, Junhong Chen, Kai Liu, Huqiang Dong, Xiaocong Li

**Affiliations:** ^1^ Department of Hepatobiliary and Pancreatic Surgery, General Surgery Center, The First Hospital of Jilin University, Changchun, 130021, Jilin, China, jlu.edu.cn; ^2^ School of Public Health, Ningxia Medical University, Yinchuan, 750004, Ningxia, China, nxmu.edu.cn; ^3^ Department of Pharmacy, China-Japan Friendship Hospital, Beijing, 100020, China, zryhyy.com.cn; ^4^ Clinical Trial Research Center, China-Japan Friendship Hospital, Beijing, 100020, China, zryhyy.com.cn

**Keywords:** gallstone disease, HDL-related inflammatory indices, lymphocyte-to-high-density lipoprotein cholesterol ratio, monocyte-to-high-density lipoprotein cholesterol ratio, neutrophil-to-high-density lipoprotein cholesterol ratio, NHANES, platelet-to-high-density lipoprotein cholesterol ratio

## Abstract

**Background:**

Gallstone disease is a condition affecting the digestive system, strongly linked to inflammation and lipid metabolism. Inflammatory markers derived from high‐density lipoprotein (HDL), incorporating both immune cells and HDL‐C, play a crucial role in assessing inflammatory responses. This study aims to explore the relationship between these HDL‐related inflammatory indices and gallstone disease.

**Methods:**

The study population was derived from the National Health and Nutrition Examination Survey (NHANES) 2017–2020 and 2021–2023 datasets. To assess the association between HDL‐related inflammatory indices and gallstone disease, weighted multivariable logistic regression and restricted cubic spline (RCS) analysis were utilized. Additionally, subgroup analysis was conducted to confirm the consistency of the results across different subpopulations.

**Results:**

Among the 16,871 participants included in the study, 11.0% were diagnosed with gallstone disease. When compared to the lowest quartile, those in the highest quartile of lymphocyte‐to‐HDL cholesterol ratio (LHR), monocyte‐to‐HDL cholesterol ratio (MHR), neutrophil‐to‐HDL cholesterol ratio (NHR), and platelet‐to‐HDL cholesterol ratio (PHR) faced an elevated risk of gallstone disease by 58.6% (OR = 1.586, 95% CI: 1.143–2.2), 67.6% (OR = 1.676, 95% CI: 1.275–2.204), 68.7% (OR = 1.687, 95% CI: 1.244–2.287), and 42.7% (OR = 1.427, 95% CI: 1.101–1.849), respectively. The correlation between HDL‐related inflammatory indices and gallstone disease was more pronounced in females, individuals without diabetes or hypertension, nonsmokers, and those who consumed alcohol.

**Conclusions:**

This research identified a positive correlation between HDL‐related inflammatory indices and gallstone disease in a nationally representative sample. These indices can be derived from routine blood tests at no additional cost, making them practical and cost‐effective tools for early risk stratification and potential large‐scale screening.

## 1. Introduction

Gallstone disease is a prevalent hepatobiliary disease, with a global prevalence of approximately 6% [1]. Over the past 30 years, the prevalence of gallstone disease among the U.S. population has increased from 7.4% to 13.9%, accompanied by a doubling in the proportion of individuals undergoing gallbladder surgery [2]. The healthcare visits and expenditures associated with gallstone disease impose a substantial burden on both clinical practice and public health [3]. Gallstones are categorized into cholesterol, pigment, and mixed types, with cholesterol stones being the most common [4]. Their development is driven by the supersaturation of cholesterol and calcium salts in bile, leading to cholesterol crystallization [5]. Several factors contribute to gallstone disease risk, including age, gender, genetic susceptibility, diet, and lifestyle [4]. Additionally, inflammatory responses and lipid metabolism disorders contribute to gallstone formation [6, 7]. Therefore, investigating clinical indicators that comprehensively reflect inflammatory status and lipid metabolism is of significant public health importance for enhancing gallstone disease prevention strategies.

Traditional inflammatory markers such as high‐sensitivity C‐reactive protein (hs‐CRP) are widely used to assess systemic inflammation, but they primarily reflect hepatic acute‐phase responses and may not fully capture immune–lipid interactions [8, 9]. In contrast, high‐density lipoprotein (HDL) plays a dual role, not only in reverse cholesterol transport but also in exerting anti‐inflammatory and antioxidant effects, including modulating cytokine production and immune cell activation [10, 11]. In addition to promoting cholesterol efflux via ABCA1/ABCG1, HDL inhibits monocyte/macrophage activation and reduces proinflammatory cytokine release [12, 13]. HDL influences neutrophil extracellular trap (NET) formation and platelet–leukocyte interactions, processes implicated in biliary inflammation and crystal nucleation [14, 15]. Emerging evidence suggests that immune cell counts—such as lymphocytes, monocytes, neutrophils, and platelets—are strongly associated with systemic inflammation and metabolic diseases [16, 17]. The complex interactions between HDL these immune cells provides the biological basis for novel HDL‐related inflammatory indices, including lymphocyte‐to‐HDL cholesterol ratio (LHR), monocyte‐to‐HDL cholesterol ratio (MHR), neutrophil‐to‐HDL cholesterol ratio (NHR), and platelet‐to‐HDL cholesterol ratio (PHR) [18–20]. These indices simultaneously integrate inflammatory and lipid metabolic status, offering a more comprehensive reflection of the inflammatory–lipid axis compared to single biomarkers like hs‐CRP. Given that gallstone disease arises from both cholesterol supersaturation and inflammatory processes, HDL‐related inflammatory indices may serve as more precise and cost‐effective markers for identifying individuals at elevated risk [21, 22]. Prior research has investigated the relationship between these indices and conditions such as periodontitis, Parkinson’s disease, and metabolic syndrome [23–25]. In addition, the study by Ma et al. [26] also found that HDL‐related inflammatory indices can be used as potential biomarkers for occupational mental health assessment. However, their association with gallstone disease has not been comprehensively investigated.

This study aims to examine the association between HDL‐related inflammatory indices and gallstone disease using data from the National Health and Nutrition Examination Survey (NHANES).

## 2. Materials and Methods

### 2.1. Study Design

NHANES is structured to evaluate the health and nutritional status of the U.S. population through a combination of interviews, physical assessments, and laboratory analyses. The survey was reviewed and approved by the National Center for Health Statistics Ethics Review Board and obtained written informed consent from all participants.

**Figure 1:**
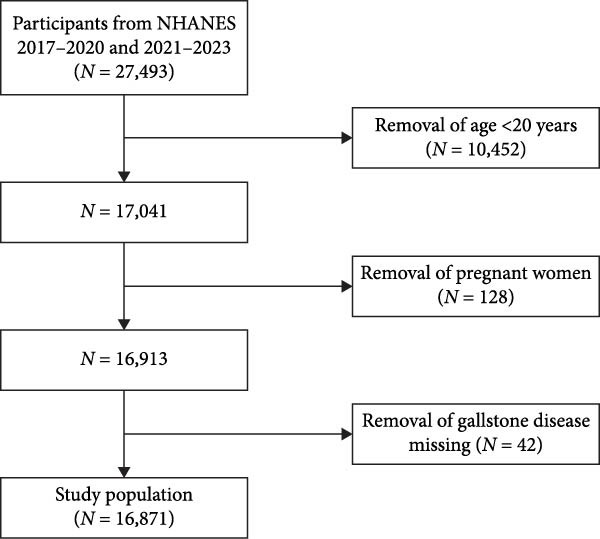
Flowchart for study population.

This research analyzed data from the two most recent survey cycles (2017–March 2020 and August 2021–August 2023), encompassing a total of 27,493 participants. The exclusion criteria were: (1) individuals younger than 20 years; (2) pregnant women; (3) participants with missing responses on the gallstone questionnaire. The final study population consisted of 16,871 individuals (Figure 1).

### 2.2. Study Variables

The exposure variables in this study were HDL‐related inflammatory indices, including LHR, MHR, NHR, and PHR. These indices were calculated using the following formulas: LHR = lymphocyte count/HDL‐C, MHR = monocyte count/HDL‐C, NHR = neutrophil count/HDL‐C, PHR = platelet count/HDL‐C. The outcome variable was the presence of gallstones.

Covariates included age, gender, race, poverty‐income ratio (PIR), education level, marital status, smoking and drinking status, hypertension, and diabetes. Smoking status was determined based on participants’ responses to the question “Have you smoked at least 100 cigarettes in your lifetime?” Alcohol consumption status was determined according to responses to the question “Have you had at least 12 alcoholic drinks in any 1 year?” Diagnoses of diabetes and hypertension were based on participants’ responses to the question “Have you ever been told by a doctor or other health professional that you have diabetes or hypertension?” Detailed explanations, data collection methods, and calculation procedures for all study variables are available in the NHANES official website.

### 2.3. Statistical Analysis

Continuous variables were analyzed using analysis of variance (ANOVA) and Kruskal–Wallis H tests, while categorical variables were compared using chi‐square tests. To evaluate the relationship between LHR, MHR, NHR, PHR, and gallstone disease, these indices were incorporated into a weighted multivariable logistic regression model, first as continuous variables and then as quartiles. Restricted cubic splines (RCSs) with four knots were employed to visualize the associations between LHR, MHR, NHR, PHR, and gallstone disease. Additionally, subgroup analyses were conducted across different populations to validate the robustness of the findings. All statistical analyses were performed using R Studio 4.2.2, with a two‐sided *p*‐value below 0.05 considered statistically significant.

## 3. Results

### 3.1. Baseline Characteristics of the Participants

This study included a total of 16,871 participants, of whom 8919 were female, accounting for 52.9% of the population. Among the study population, 1850 individuals were diagnosed with gallstones, yielding a prevalence rate of 11.0%. Participants with gallstones were generally older, had a higher BMI, and were more likely to be female, non‐Hispanic White, smokers, nondrinkers, and individuals with diabetes and hypertension (Table 1). Furthermore, participants with gallstone disease exhibited higher HDL‐related inflammatory indices (*p*  < 0.05).

**Table 1:** Basic characteristics of the participants.

Characteristics	Total (*N* = 16,871)	Without gallstones (*N* = 15,021)	With gallstones (*N* = 1850)	*p*‐Value
Age (year), mean ± SD	52.4 ± 17.6	51.5 ± 17.6	59.9 ± 15.5	<0.0001
Gender, *n* (%)	—	—	—	<0.0001
Male	7952 (47.1)	7437 (49.5)	515 (27.8)	—
Female	8919 (52.9)	7584 (50.5)	1335 (72.2)	—
Race, *n* (%)	—	—	—	<0.0001
Mexican American	1584 (9.4)	1408 (9.4)	176 (9.5)	—
Other Hispanic	1690 (10.0)	1481 (9.9)	209 (11.3)	—
Non‐Hispanic White	7711 (45.7)	6738 (44.9)	973 (52.6)	—
Non‐Hispanic Black	3412 (20.2)	3126 (20.8)	286 (15.5)	—
Other race	2474 (14.7)	2268 (15.1)	206 (11.1)	—
Education level, *n* (%)	—	—	—	0.1125
Less than high school	2768 (16.4)	2444 (16.3)	324 (17.5)	—
High school	3940 (23.4)	3486 (23.3)	454 (24.6)	—
More than high school	10,127 (60.2)	9056 (60.4)	1071 (57.9)	—
Marital status, *n* (%)	—	—	—	0.6731
Cohabitation	9308 (55.3)	8295 (55.3)	1013 (54.8)	—
Solitude	7531 (44.7)	6696 (44.7)	835 (45.2)	—
PIR, mean ± SD	2.7 ± 1.6	2.8 ± 1.7	2.6 ± 1.6	< 0.0001
Smoking status, *n* (%)	—	—	—	0.0006
Yes	7034 (41.7)	6186 (41.2)	848 (45.8)	—
No	9806 (58.1)	8808 (58.6)	998 (53.9)	—
Drinking status, n (%)	—	—	—	< 0.0001
Yes	6318 (53.2)	5804 (54.8)	514 (40.2)	—
No	5559 (46.8)	4793 (45.2)	766 (59.8)	—
Diabetes, n (%)	—	—	—	< 0.0001
Yes	2473 (14.7)	2019 (13.4)	454 (24.5)	—
No	14,393 (85.3)	12,997 (86.5)	1396 (75.5)	—
Hypertension, n (%)	—	—	—	< 0.0001
Yes	6477 (38.4)	5459 (36.3)	1018 (55.0)	—
No	10,371 (61.5)	9540 (63.5)	831 (44.9)	—
BMI (kg/m^2^), mean ± SD	29.9 ± 7.5	29.5 ± 7.2	33.4 ± 8.6	< 0.0001
LHR, mean ± SD	1.7 ± 3.2	1.7 ± 1.1	1.9 ± 9.4	0.0274
MHR, mean ± SD	0.4 ± 0.2	0.4 ± 0.2	0.5 ± 0.3	0.0059
NHR, mean ± SD	3.3 ± 1.7	3.2 ± 1.7	3.5 ± 2.0	< 0.0001
PHR, mean ± SD	193.3 ± 78.0	192.6 ± 76.1	198.8 ± 92.3	0.0383

*Note*: LHR, lymphocyte‐to‐high‐density lipoprotein cholesterol ratio; MHR, monocyte‐to‐high‐density lipoprotein cholesterol ratio; NHR, neutrophil to high‐density lipoprotein cholesterol ratio; PHR, platelet‐to‐high‐density lipoprotein cholesterol ratio; PIR, the ratio of income to poverty.

Abbreviation: BMI, body mass index.

### 3.2. Associations Between HDL‐Related Inflammatory Indices and Gallstone Disease

Table 2 presents the association between HDL‐related inflammatory indices and gallstone disease. In Model 3, each unit increase in MHR, NHR, and PHR was linked to a 77.8% (OR = 1.778, 95% CI: 1.224–2.583), 9.3% (OR = 1.093, 95% CI: 1.035–1.154), and 0.2% (OR = 1.002, 95% CI: 1.001–1.003) higher likelihood of developing gallstone disease, respectively. When HDL‐related inflammatory indices were analyzed as categorical variables, participants in the highest quartile of LHR, MHR, NHR, and PHR faced an elevated risk of gallstone disease by 58.6%, 67.6%, 68.7%, and 42.7%, respectively, compared to those in the lowest quartile.

**Table 2:** Association between high‐density lipoprotein‐related inflammatory indices and gallstone disease.

Exposure	Model 1 OR (95% CI)		Model 2 OR (95% CI)	Model 3 OR (95% CI)
LHR	1.035 (0.97,1.105)		1.178 (0.926,1.498)	1.055 (0.965,1.154)
LHR (quartile)				
Q1	1.00 (Reference)		1.00 (Reference)	1.00 (Reference)
Q2	1.123 (0.953,1.324)		1.463 (1.24,1.726)	1.216 (0.983,1.506)
Q3	1.136 (0.923,1.398)		1.697 (1.377,2.091)	1.47 (1.129,1.914)
Q4	1.19 (0.919,1.542)		2.135 (1.609,2.834)	1.586 (1.143,2.2)
*p*‐Value for trend	0.261		< 0.001	0.008
MHR	1.404 (1.009,1.952)		2.536 (1.774,3.624)	1.778 (1.224,2.583)
MHR (quartile)				
Q1	1.00 (Reference)		1.00 (Reference)	1.00 (Reference)
Q2	1.246 (0.979,1.586)		1.502 (1.171,1.926)	1.421 (1.056,1.911)
Q3	1.327 (1.096,1.606)		1.799 (1.472,2.198)	1.533 (1.196,1.965)
Q4	1.181 (0.945,1.477)		1.915 (1.524,2.404)	1.676 (1.275,2.204)
*p*‐Value for trend	0.164		< 0.001	< 0.001
NHR	1.097 (1.043,1.153)		1.172 (1.118,1.23)	1.093 (1.035,1.154)
NHR (quartile)				
Q1	1.00 (Reference)		1.00 (Reference)	1.00 (Reference)
Q2	1.234 (0.921,1.652)		1.421 (1.034,1.952)	1.334 (0.961,1.85)
Q3	1.392 (1.154,1.678)		1.717 (1.408,2.094)	1.504 (1.192,1.898)
Q4	1.595 (1.242,2.049)		2.266 (1.761,2.915)	1.687 (1.244,2.287)
*p*‐Value for trend	< 0.001		< 0.001	0.001
PHR	1.001 (1,1.002)		1.003 (1.002,1.004)	1.002 (1.001,1.003)
PHR (quartile)				
Q1	1.00 (Reference)		1.00 (Reference)	1.00 (Reference)
Q2	0.969 (0.764,1.228)		1.209 (0.953,1.534)	1.164 (0.879,1.541)
Q3	1.035 (0.804,1.332)		1.459 (1.109,1.918)	1.206 (0.894,1.627)
Q4	1.153 (0.917,1.449)		1.83 (1.449,2.311)	1.427 (1.101,1.849)
*p*‐Value for trend	0.183		< 0.001	0.015

*Note*: Model 1: no covariates were adjusted. Model 2: age, gender, and race were adjusted. Model 3: age, gender, race, education level, marital status, PIR, diabetes, hypertension, and smoking and drinking status were adjusted.

To gain deeper insight into the relationship between these indices and gallstone disease, RCS curves were applied (Figure 2). The analysis demonstrated a nonlinear upward trend in gallstone disease risk as MHR levels increased. The risk of gallstone disease initially increased and then stabilized as LHR and NHR levels rose. In contrast, an increase in PHR resulted in slight fluctuations in gallstone disease risk.

Figure 2The association between LHR, MHR, NHR, PHR, and gallstone disease. (A) Association between LHR and gallstone disease. (B) Association between MHR and gallstone disease. (C) Association between NHR and gallstone disease. (D) Association between PHR and gallstone disease. LHR, lymphocyte‐to‐high‐density lipoprotein cholesterol ratio; MHR, monocyte‐to‐high‐density lipoprotein cholesterol ratio; NHR, neutrophil to high‐density lipoprotein cholesterol ratio; PHR, platelet‐to‐high‐density lipoprotein cholesterol ratio.

**Figure:**
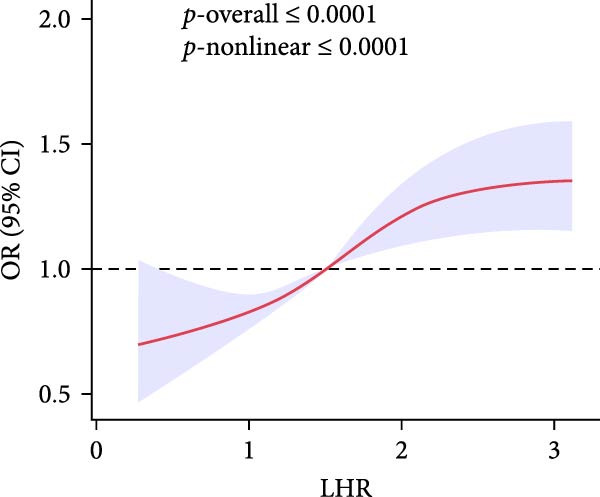
(A)

**Figure:**
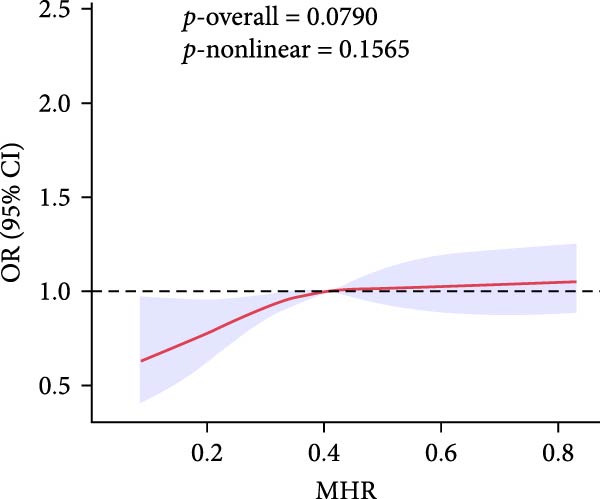
(B)

**Figure:**
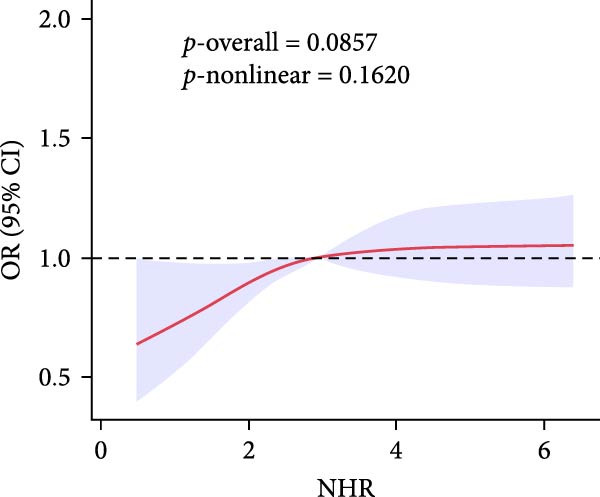
(C)

**Figure:**
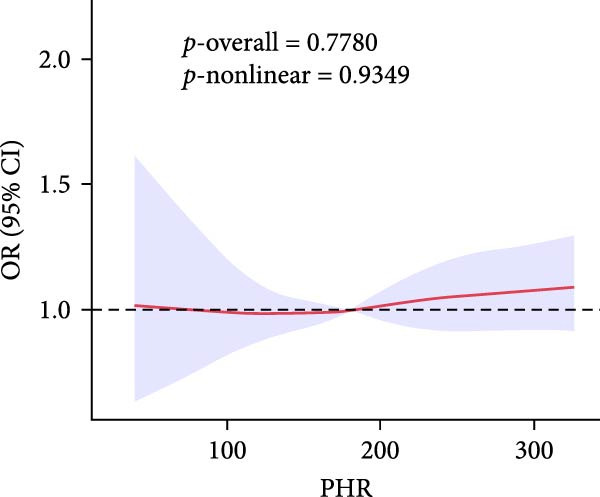
(D)

### 3.3. Subgroup Analyses

**Figure 3:**
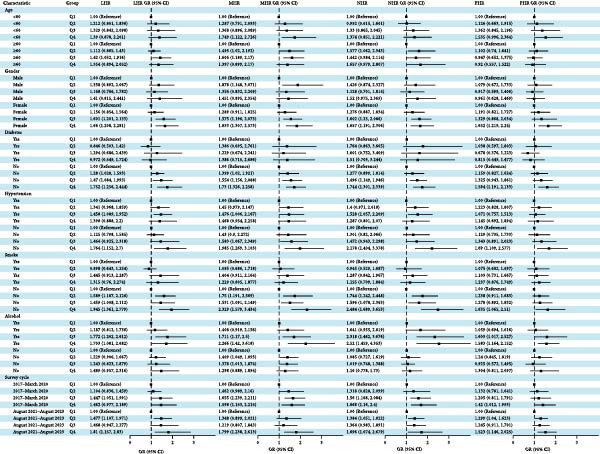
Subgroup analysis of the relationship between LHR, MHR, NHR, PHR, and gallstone disease.

To investigate the relationship between these inflammatory indices and gallstone disease across various populations, a subgroup analysis was conducted. As shown in Figure 3, the positive associations between these indices and gallstone disease were more pronounced among females, individuals without diabetes or hypertension, nonsmokers, and those who consumed alcohol. Specifically, among females, the risk of gallstone disease in the fourth quartile groups of LHR, MHR, NHR, and PHR increased by 66%, 83.3%, 65.7%, and 65.2%, respectively, compared with those in the first quartile group. Among participants without diabetes, the risks increased by 75.2%, 73%, 74.4%, and 59.4%, respectively. Among participants without hypertension, the risks increased by 76.4%, 98.5%, 117.8%, and 69%, respectively. In nonsmokers, the risks increased by 94.5%, 132.9%, 148.4%, and 63.5%, respectively. In alcohol drinkers, the risks increased by 70.3%, 126.6%, 152%, and 58.3%, respectively. Stratified analyses by survey cycle showed that associations between HDL‐related inflammatory indices and gallstone disease were consistent across the 2017–2020 (pre‐COVID) and 2021–2023 (post‐COVID) cycles.

## 4. Discussion

This study contributes by identifying a positive association between HDL‐related inflammatory indices and gallstone disease in a nationally representative sample. Further subgroup analysis indicated that this relationship was more prominent among females, nonsmokers, alcohol consumers, and individuals without diabetes or hypertension. These findings underscore the potential utility of HDL‐related inflammatory indices—which integrate inflammatory cell counts with HDL‐C and can be calculated from routine laboratory tests—as low‐cost tools for risk stratification in clinical practice. In practical terms, such indices could be implemented via electronic health record (EHR) automation to flag individuals in the upper distribution for targeted prevention: (1) targeted lifestyle interventions; (2) optimization of metabolic conditions (improved glycemic, lipid, and blood‐pressure control); (3) selective imaging surveillance for individuals with persistently elevated indices. Overall, HDL‐related indices may aid early risk identification and guide targeted preventive efforts, but their routine clinical adoption should follow confirmatory prospective studies and implementation research.

Previous research has examined the relationship between inflammatory markers, including C‐reactive protein (CRP) and complete blood cell count‐derived indices, and the risk of gallstones. A prospective cohort study involving 95,319 participants revealed that elevated high‐sensitivity CRP levels were linked to an increased likelihood of gallstone disease [27]. Additionally, inflammatory indices integrating neutrophil, monocyte, lymphocyte, and platelet counts have been validated as predictors of gallstone risk in U.S. adults under the age of 60 [28]. Liu et al. [29] found that circulating inflammatory proteins were associated with gallstones. Moreover, lipid metabolism, a significant factor in gallstone development, has been extensively studied, particularly regarding its key components and related biomarkers. A study integrating a multicenter cross‐sectional analysis with a meta‐analysis thoroughly assessed the link between lipid metabolism and gallstone risk, identifying an inverse relationship between HDL‐C and gallstone prevalence [7]. Research by Wang et al. [30] further confirmed the association between lipid profiles and gallstone disease, highlighting total cholesterol (TC), triglycerides (TG), low‐density lipoprotein cholesterol (LDL), and apolipoprotein B (APOB) as risk factors, while HDL emerged as a protective factor. Given the predictive value of inflammation‐ and lipid metabolism‐related biomarkers in assessing gallstone disease risk, exploring the connection between HDL‐related inflammatory indices and gallstones may offer more comprehensive insights.

The mechanisms by which inflammation and immune cells contribute to gallstone formation involve several key processes. First, the decondensation of chromatin during NET formation plays a pivotal role in triggering and promoting gallstone development [31, 32]. Upon encountering calcium or cholesterol crystals, neutrophils undergo chromatin decondensation and form NETs, which subsequently aggregate bile calcium and cholesterol crystals, facilitating gallstone formation [33, 34]. Second, platelets play a critical role in the inflammatory response by amplifying inflammation through P‐selectin, CD40L, and microparticles, and by closely interacting with leukocytes [35, 36]. During inflammation, platelets interact with neutrophils and lymphocytes, thereby enhancing monocyte adhesion, migration, and the release of various inflammatory mediators [37]. Inflammation induces histopathological changes in the gallbladder wall, altering its contractility and epithelial transport capacity [38, 39]. Dysfunction in gallbladder motility impairs bile emptying and contraction, thereby promoting cholesterol nucleation and gallstone formation [40, 41]. Third, neutrophils, monocytes, and lymphocytes produce reactive oxygen species (ROS) both intracellularly and extracellularly, leading to cellular redox imbalance and oxidative stress [42]. Oxidative stress contributes to protein and lipid peroxidation, reducing the activity of key enzymes involved in cholesterol and bilirubin metabolism, ultimately facilitating gallstone development [43]. Additionally, HDL exerts anti‐inflammatory effects by inhibiting the production of cytokines and chemokines in monocytes, macrophages, and monocyte‐derived dendritic cells. HDL also downregulates the expression of costimulatory molecules and antigen presentation, further modulating the inflammatory response [44].

Our subgroup analyses showed stronger positive associations of LHR, MHR, NHR, and PHR with gallstone disease among females, nonsmokers, alcohol consumers, and participants without diabetes or hypertension. Several mechanisms may explain these patterns. First, sex hormones—notably estrogens—increase hepatic biliary cholesterol secretion, alter bile composition and modulate gallbladder motility; estrogens also influence lipid metabolism and immune responses, which plausibly amplify relationships between HDL‐related inflammatory indices and gallstone formation in females [45]. Second, participants with chronic metabolic comorbidities commonly exhibit persistent low‐grade inflammation, altered HDL functionality, and frequent use of medications that modify lipid profiles [46]. Such baseline alterations or therapeutic effects may attenuate the incremental signal of HDL‐derived inflammatory ratios; conversely, in participants without these comorbidities, elevations in these indices may more specifically reflect early pathophysiologic processes linked to gallstone risk. Third, smoking elevates systemic inflammatory markers and impairs HDL functional properties; smokers, therefore, may show higher background inflammatory measures, reducing the discriminatory power of HDL‐related indices [47]. This could explain why associations were more evident among nonsmokers in our sample. Fourth, alcohol exerts complex effects: moderate alcohol intake is often associated with higher HDL‐C and some studies report inverse associations with gallstone disease, whereas heavy or specific drinking behaviors may have different effects [48]. Heterogeneity in drinking intensity and patterns, possible interactions with HDL metabolism, and alcohol‐related changes in bile composition or gut microbiota could lead to stronger observed associations among self‐reported drinkers in our data. Overall, these subgroup findings suggest that the HDL‐inflammation axis may operate differently across population strata, and that clinical utility of HDL‐related indices may be context dependent.

The strength of this study lies in its rigorous survey design and the nationally representative sample population. Additionally, HDL‐related inflammatory indices are easily obtainable and cost‐effective. Compared to inflammatory indices derived solely from complete blood cell counts, HDL‐related inflammatory indices offer a more comprehensive perspective by integrating both inflammatory and lipid metabolic components, enhancing their predictive value for gallstone disease. However, several limitations should be acknowledged. First, although extensive covariate adjustments were made, we were unable to perform comprehensive sensitivity analyses for lipid‐lowering medications. Second, as some data were collected through self‐reported questionnaires, there is a risk of recall bias. Third, the survey design prevents the establishment of causality, necessitating longitudinal prospective research to validate the observed associations.

## 5. Conclusion

This study highlights a significant association between elevated HDL‐related inflammatory indices and an increased risk of gallstone disease among U.S. adults. Further prospective research is necessary to clarify the underlying mechanisms and determine a causal link between these inflammatory indices and gallstone disease.

## Ethics Statement

The protocol of NHANES has been approved by the Ethical Review Board of the National Center for Health Statistics, and the participants furnished written informed consent before participation.

## Consent

The authors have nothing to report.

## Conflicts of Interest

The authors declare no conflicts of interest.

## Author Contributions

Conceptualization, methodology: Chang Fu and Xiaocong Li. software: Huqiang Dong. Validation, visualization: Junhong Chen. Formal analysis: Xiaocong Li and Huqiang Dong. Investigation, supervision, project administration: Kai Liu. Data curation: Chang Fu and Junhong Chen. Writing – original draft preparation: Chang Fu. Writing – review and editing: Xiaocong Li. All the authors have contributed to the study conception and design.

## Funding

This study was supported by the National High Level Hospital Clinical Research Funding and Elite Medical Professionals Initiative of China‐Japan Friendship Hospital (NO. ZRJY2025‐QMPY11).

## Data Availability

The data that support the findings of this study are openly available in NHANES at https://www.cdc.gov/nchs/nhanes/.
